# Diversity of Astroviruses Circulating in Humans, Bats, and Wild Birds in Egypt

**DOI:** 10.3390/v12050485

**Published:** 2020-04-26

**Authors:** Ahmed El Taweel, Ahmed Kandeil, Ahmed Barakat, Omar Alfaroq Rabiee, Ghazi Kayali, Mohamed Ahmed Ali

**Affiliations:** 1 Center of Scientific Excellence for Influenza Virus, Environmental Research Division, National Research Centre, El-Buhouth Street, Dokki, Giza 12311, Egypt; ahmed.nageh@human-link.org (A.E.T.); kandeil_a@hotmail.com (A.K.); 2 Microbiology Department, Faculty of Science, Ain Shams University, Cairo 11566, Egypt; dr.barakat51@hotmail.com (A.B.); Omaralfarouk2000@yahoo.com (O.A.R.); 3 Human Link, Hazmieh 1109, Lebanon; 4 Department of Epidemiology, Human Genetics, and Environmental Sciences, University of Texas, Houston, TX 77030, USA

**Keywords:** Astroviruses, bat viruses, Egypt, Mamastroviruses, Avastroviruses

## Abstract

Astroviruses belong to *Astroviridae* family which includes two main genera: Mamastroviruses that infect mammals, and Avastroviruses that infect avian hosts. Bats and wild birds are considered among the natural reservoirs for astroviruses. Infections in humans are associated with severe gastroenteritis, especially among children. We conducted surveillance for astroviruses in bats, wild birds, and humans in Egypt. Our results indicated relatively high prevalence of astroviruses in those hosts. Phylogenetic analysis revealed diversity of these viruses within hosts. Detected human viruses showed similarity with classic and variant human astroviruses, as well as similarity with animal-origin viruses. Viruses in bats were dispersed, with similarities to other bat viruses as well as other mammalian, including human, viruses. Wild bird viruses varied and were related to other avastroviruses, as well as human astroviruses. Our results indicate that astroviruses are common in bats, wild birds, and humans in Egypt, with a wide gene pool. Potential cross-species transmission may be occurring but should be verified by further surveillance and molecular studies.

## 1. Introduction

Astroviruses are small icosahedral non-enveloped viruses with positive-sense, single-stranded RNA genome. They are 6.4 to 7.3 kb long and consist of three open reading frames (ORF1a, ORF1b, and ORF2). Astroviruses were first detected in stool samples of humans in 1975, then subsequently identified in a wide variety of mammalian and avian host species [1,2]. Astroviruses belong to the *Astroviridae* family that includes two main genera: Mamastroviruses that infect mammals, and Avastroviruses that infect avian hosts [3]. Genetic evolution analysis of previously detected astroviruses showed the ability of astroviruses to increase their host range by infecting a new host [4]. In most infected hosts, astroviruses cause gastroenteritis, but some avian astroviruses have been associated with both intestinal and extra-intestinal symptoms [5]. Human astroviruses are one of the causative agents of gastroenteritis in humans and other mammals [6]. The main transmission route of human astroviruses is the fecal-oral route.

Wildlife plays a major role in emerging zoonotic diseases, especially viral infections. Certain animals, such as bats and birds, are known to be reservoirs for many zoonotic pathogens [7]. Therefore, surveillance for zoonotic viruses in those wildlife hosts is likely to enhance our knowledge of circulating viruses and disease emergence potential prior to spillover to people [8]. Several studies showed high prevalence rates and important genetic diversity of astroviruses in bats sampled in China, Korea, Lao PDR, Cambodia, Italy, Germany, Hungary, Mozambique, and Gabon [9,10,11,12,13,14]. In Egypt, several species of bats exist. *Rousettus aegyptiacus* (Fruit bat) is the most common bat species in Egypt, residing in abandoned structures and fruit gardens in close contact with humans, indicating a probable transmission potential of zoonotic viruses.

The wetlands of the northern Nile Delta of Egypt serve as a vital stopover for millions of migratory birds during their annual migration between the Palearctic and Afrotropical ecozones [15]. Therefore, the Egyptian environment is an important site on the migration network of wild birds through the old world [16]. In addition to migratory birds, Egypt has several species of resident birds that live in close contact with humans and might play a role in the transmission of astroviruses.

Until now, no data was available on the genetic diversity of circulating astroviruses in wild birds, bats, and humans in Egypt. In this context, studies on the prevalence and phylogenetic relationships of contemporary circulating astroviruses among wildlife, including bats, wild birds, and humans, are needed to better understand the ecology and zoonosis of these viruses in Egypt.

## 2. Materials and Methods

### 2.1. Sample Collection

#### 2.1.1. Human Samples

A total of 100 human stool samples were collected from children less than 5 years of age, admitted to two major general hospitals in Cairo, and diagnosed with long term diarrhea during the winter season of 2016/2017. Fresh fecal samples were diluted by a factor of 10^−1^ in viral transport medium (1 gm/10 mL) and transported on ice for further laboratory processing.

#### 2.1.2. Bat Samples

During the period from May to October 2016, a total of 417 rectal swabs were collected from healthy bats of four different species (*Rousettus aegyptiacus* (*n* = 288), *Pipistrellus aegyptius* (93), *Nycteris thebaica* (25), and *Taphozous perforates* (11)) captured from Giza (301) and Damietta (116) governorates. Bats were captured from caves as well as abandoned mudbrick houses using mist nets and were characterized by a taxonomist. Bats were released at the capturing site after sampling. The collected swabs were immersed in viral transport medium and transported on ice rapidly, for further laboratory processing.

#### 2.1.3. Migratory Bird Samples

From October to December 2016, a total of 301 cloacal swabs were collected from 301 birds of eighteen different species of wild birds, including resident (*n* = 7) and migratory (*n* = 12) birds captured from five governorates (Aswan (79), Fayoum (19), Damietta (113), Port Said (66), and Marsa Matruh (24)) in Egypt. All birds were characterized by a taxonomist. Samples were collected on viral transport medium, then transferred in ice boxes to the laboratory for processing. All captured birds were released at the site of sampling.

### 2.2. Detection of Astrovirus in Bats and Humans

A volume of 140 µL of each sample was subjected to viral RNA extraction using a QIAamp virus RNA mini kit (Qiagen, Hilden, Germany). RNA was subjected to the synthesis of cDNA by using a RevertAid First Strand cDNA Synthesis Kit (Thermo Scientific, Waltham, MA, USA). Reactions were initiated by incubation at 65 °C for 5 min, followed by 60 min at 42 °C, and terminated by heating at 70 °C for 5 min. For the first round of nested PCR for the detection of astroviruses in bats and humans, 2 µL of each specimen’s cDNA was mixed with 12.5 µL Green GoTaq master mix (Promega, Madison, WI, USA), and 50 pmol of forward (forward primers, 5′-GARTTYGATTGGRCKCGKTAYGA-3′ and 5′-GARTTYGATTGGRCKAGGTAYGA-3′) and reverse primer (5′-GGYTTKACCCACATNCCRAA-3′) [17]. After an initial incubation at 95 °C for 1 min, 30 cycles of amplification were carried out, consisting of denaturation at 95 °C for 30 s, annealing at 50 °C for 30 s, and extension at 68 °C for 30 s. Hemi-nested PCR was carried out with a mixture of two forward primers, 5′-CGKTAYGATGGKACKATHCC-3′ and 5′-AGGTAYGATGGKACKATHCC-3′, and the same reverse primer used in the first-round PCR; the thermocycling conditions were the same as those used for the first-round PCR, except that 40 cycles of amplification were performed. PCR products were analyzed by standard agarose gel electrophoresis. The expected product size of the second PCR band was 422 bp. All positive results were verified by direct DNA sequencing of the PCR amplicons.

### 2.3. Detection of Astroviruses in Wild Birds

Degenerate primers were designed to target conserved regions in the RNA-dependent-RNA polymerase (RdRp) [18]. RNA was extracted from collected samples of migratory birds using the QIAamp Viral RNA Mini Kit (Qiagen). Reverse transcription PCR for the RdRp was performed using the OneStep RT-PCR Kit (Qiagen) according to standard protocol using forward primer Astr4380F (5′-GAYTGGRCNCGNTWYGATGGNACIAT-3′) and reverse primer Astr4811R (5′-GGYTTNACCCACATNCCAAA-3′), with a reverse transcription at 50 °C for 30 min and initial PCR activation at 94 °C for 5 min, followed by 36 cycles of denaturation at 94 °C for 30 s, annealing at 45 °C for 60 s, DNA extension at 72 °C for 60 s, and a final extension step at 72 °C for 10 min. To increase sensitivity, a hemi-nested second round of PCR amplification was run using 2 µL of product from the first reaction in a 20 µL reaction mixture with forward primer Astr4380F and reverse primer Astr4722R (5′-ARNCKRTCATCNCCATA-3′) and polymerases (Qiagen Fast Cycling PCR Kit, Cat. No.203745). The mixtures were amplified with an initial denaturation at 95 °C for 5 min, followed by 45 cycles of denaturation at 96 °C for 8 s, annealing at 45 °C for 8 s, DNA extension at 68 °C for 12 s, and a final extension step at 72 °C for 3 min. The expected product size of the second PCR band was 342 bp.

### 2.4. Genetic and Phylogenetic Analysis of Astroviruses

The final PCR product from strong positive samples was gel purified by a PCR purification kit (Qiagen) and sequenced with the same primers at the Macrogen sequencing facility (Macrogen, Seoul, South Korea). Sequence alignment was performed using the BioEdit 7.0 software. The phylogenetic tree was elaborated using the MEGA7 program by applying the neighbor-joining method with Kimura’s two-parameter distance model and 1000 bootstrap replicates. Nucleotide pairwise distance analysis and maximum-likelihood analysis for Mamastroviruses and Avastroviruses were performed using the MEGA7 program.

### 2.5. Statistical Analysis

Statistical analyses were conducted using SPSS version 16 (IBM, Armonk, NY, USA). The association between prevalence of astrovirus in different hosts (human, bats, and wild birds) and the study variables were analyzed by Chi-square test. Statistical significance was considered at *p*-value less than 0.05.

### 2.6. Ethics Approval

The protocols for bat capture and sample collection and bird sampling were approved by the Ethics Committee at the National Research Centre (Cairo, Egypt) (Project 17-070, 3 May 2017). All applicable international, national, and/or institutional guidelines for the care and use of animals were followed. Human samples were collected as part of the regular diagnostic procedures, coded, and tested without identifying data.

## 3. Results

### 3.1. Human Samples

The patients’ symptoms included diarrhea, vomiting, abdominal pain, and high fever. Of one hundred human stool samples, 28 (28%) samples tested positive for astrovirus by hemi-nested PCR using the pan-astrovirus primers targeting the RdRp in ORF1b gene. Positivity rate in 1 year old children or younger was higher (35.21%) than in children with ages over 1 year old (10.34%) (*p*-value < 0.05). Gender-based analyses showed that no significant difference was observed between males and females (*p*-value > 0.05) (Table 1). Additionally, no significant difference was observed in the prevalence rate of astroviruses among children from different governorates in Egypt (*p*-value > 0.05).

### 3.2. Bat Samples

Prevalence rates of astroviruses in bats differed significantly by sample collection sites and type of bats (*p*-value < 0.05) (Table 1). From 417 specimens, we detected astroviruses in 119 samples (prevalence 28.53%) in all four bat species, with the highest virus detection rate in Egyptian Fruit bat (*Rousettus aegyptiacus*; 33.33%) followed by the Egyptian tomb bat (*Taphozous perforates*; 27.27 %), Egyptian slit-faced bat (*Nycteris thebaica*; 24%), and Egyptian Pipistrelle (*Pipistrellus aegyptius*; 15%). Based on sampling site, the detection rate of astroviruses in Giza (35.22%) was higher than in Damietta (11.21%; *p*-value < 0.05). There was no statistical difference in prevalence between adult and juvenile bats (*p*-value > 0.05) (Table 1).

### 3.3. Wild Birds

Out of 301 collected samples from 18 different species of wild birds, 80 samples (26.5%) were positive for astroviruses. Prevalence rates of astroviruses in wild birds differed significantly by sampling collection sites and bird species (*p*-value < 0.05) (Table 1). The astrovirus’ RNA was detected in fourteen species of birds. Based on bird species, the detection rates ranged from 11.11% to 100%. There was no statistical difference in the prevalence rate between resident and migratory wild birds (*p*-value > 0.05) (Table 1). The highest detection rate of virus RNA was in Fayoum (52.63%), followed by Mersa Matruh (16.6%), Port Said (16.6%), and Aswan (16.45%). The lowest detection rate of astroviruses was in Damietta (13.95%).

### 3.4. Phylogenetic Analysis of Bat and Human Astroviruses

**Figure 1:**
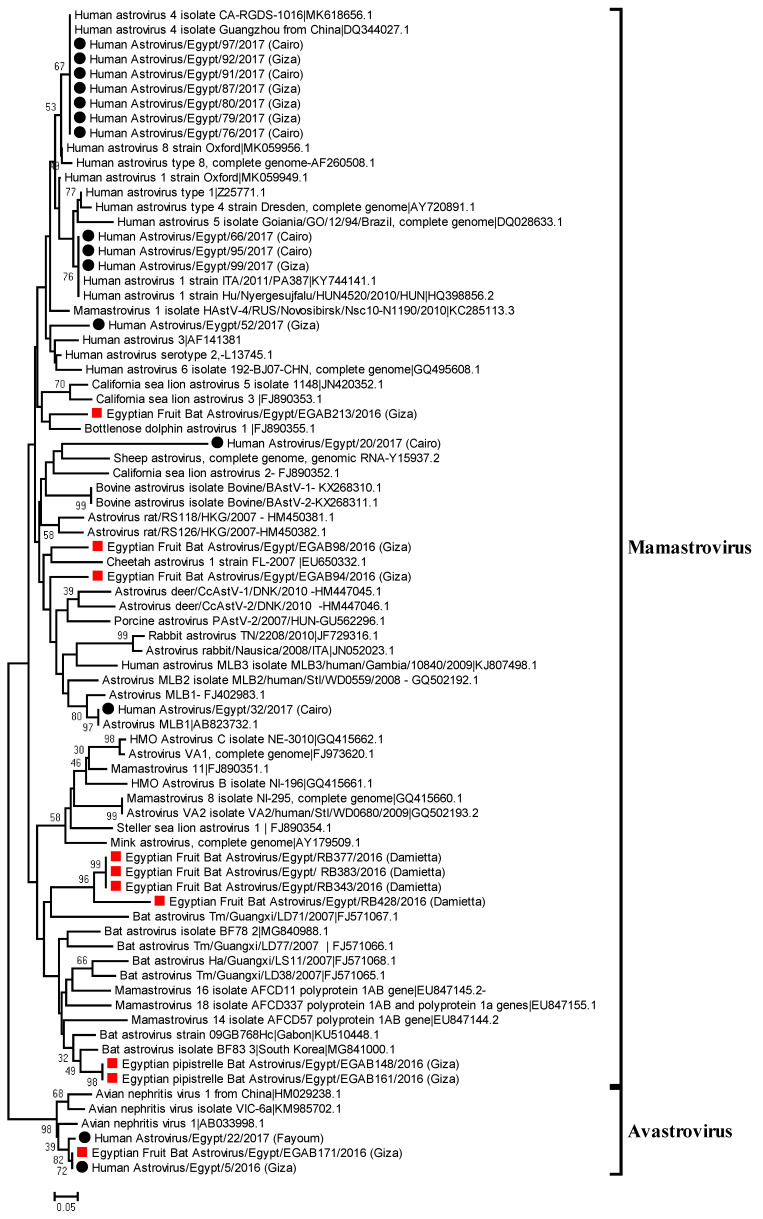
Neighbor joining phylogenetic tree of 422 bp of RNA-dependent RNA polymerase gene of the detected astroviruses in human and bat samples. Geographical sampling locations were indicated in the tree. Human and bat Astroviruses sequenced specifically for this study are labeled with black circles and red squares, respectively. The percentage of replicate trees in which the associated taxa clustered together in the bootstrap test (1000 replicates) is shown at the dendrogram nodes. The phylogenetic analysis was performed by using MEGA version 7.

A neighbor joining phylogenetic tree was constructed based on the obtained partial nucleotide sequences of RdRp from strongly positive samples (15 from humans and 10 from bats), highly similar sequences from GenBank, and reference strains of Mamastrovirus based on ICTV. The generated phylogenetic tree for RdRp nucleotide sequences showed a variety of detected human and bat astroviruses (Figure 1). Two bat astrovirus sequences from Egyptian Pipistrelle clustered with bat astroviruses detected in the common bent-wing bat and Sundevall’s roundleaf bat, in South Korea and Gabon, respectively. The tree suggested that four samples from Egyptian fruit bats were closely related to bat astrovirus, Tm/Guangxi/LD71/2007, detected in China from the black-bearded tomb bat (*Taphozous melanopogon*). One sequence from an Egyptian fruit bat was closely related to Cheetah astrovirus 1 isolated in the USA. In addition, one sequence obtained from an Egyptian fruit bat was distinct from previously detected viruses from bats, and closely related to deer astroviruses. One of the bat astroviruses detected in Egyptian fruit bats was closely related to Bottlenose dolphin astrovirus 1. When considering the astrovirus distribution between two governorates, significant bat astrovirus phylogenetic clustering was observed. These results confirm that the detected astroviruses in bats are characterized by genetic diversity and low host specificity.

Analysis of generated sequences from human positive samples indicated that seven human samples formed a cluster with a classical HAstV 4. Three sequences from human samples, clustered together with HAstV 1, were detected in gastroenteritis outbreaks in Italy and Hungary. Novel variant MLB1 HAstV was also identified in infected children. Notably, one sequence from the Egyptian fruit bat and two sequences from humans clustered together with Avian nephritis virus 1. Additionally, a positive sample from a human was phylogenetically related to sheep astrovirus.

### 3.5. Phylogenetic Analysis of Avian Astroviruses

**Figure 2:**
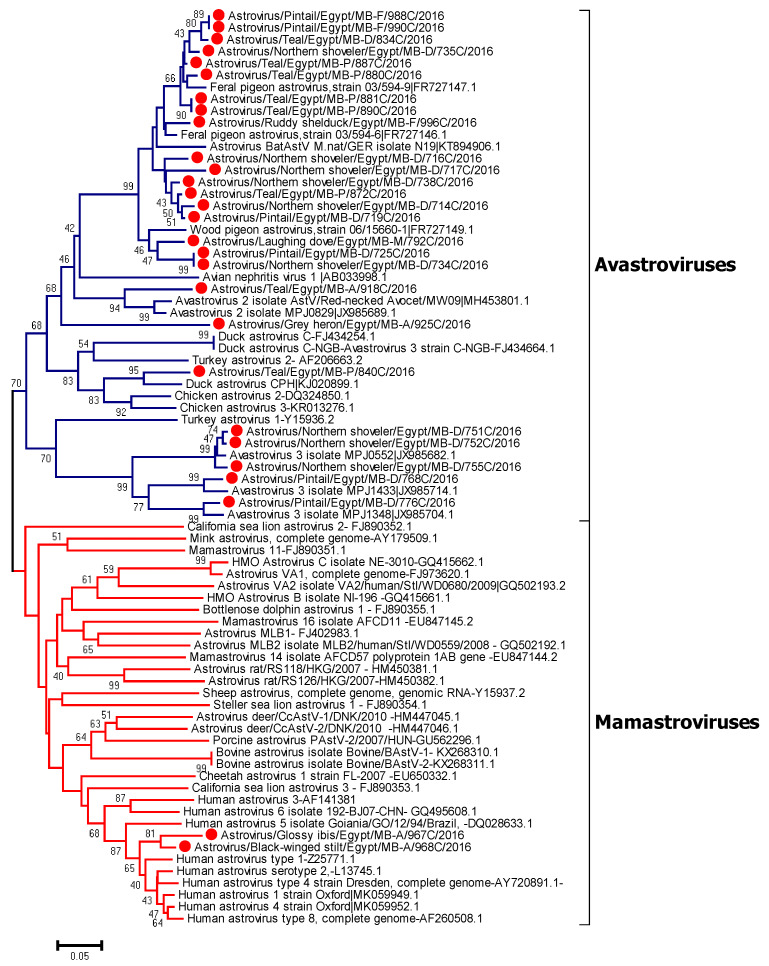
Neighbor joining phylogenetic tree of 342 bp of RNA-dependent RNA polymerase gene of the detected astroviruses in wild bird samples. Geographical sampling location was indicated in taxa of our Avastroviruses as MB-A: Aswan; MB-D: Damietta; MB-P: Port Said; and MB-F: Fayoum. Astroviruses sequenced specifically for this study are labeled with red circles. The percentage of replicate trees in which the associated taxa clustered together in the bootstrap test (1000 replicates) is shown at the dendrogram nodes. The phylogenetic analysis was performed by using MEGA version 7.

Neighbor joining phylogenetic tree was constructed based on the partial RdRp nucleotide sequences of Egyptian characterized viruses in wild birds (28 sequences), highly similar sequences retrieved from GenBank of each obtained sequence, and reference strains of Avastroviruses and Mamastrovirus based on ICTV. Analysis of generated sequences from positive samples indicated that two strains from resident birds (Black winged stilt and Glossy ibis) collected from Aswan were phylogenetically related to classical human astroviruses. The remaining avian viral sequences can be phylogenetically divided into several groups. Most of them were closely related to Feral pigeon astrovirus and Wood pigeon astrovirus. One virus detected from a teal was closely related to the virus group of Avastrovirus 2. Another isolate detected in teal was related to duck astrovirus. Some of the sequences detected from different avian hosts were found to be genetically similar. A group of detected viruses from Pintails and Northern shovelers were closely related to Avastrovirus 3, as shown in Figure 2. Maximum-likelihood analysis for Mamastroviruses and Avastroviruses were performed using the MEGA7 program to emphasize our findings, as shown in Figures S1 and S2. Pairwise nucleotide distances for Avastroviruses ranged from 0 to 1.28; while for Mamastroviruses, they ranged from 0–1.5 (Tables S1 and S2).

## 4. Discussion

While there have been no reports describing the prevalence of astrovirus in bats and wild birds in Egypt, and limited reports about children, we studied the diversity of astroviruses circulating in humans, bats, and wild birds in Egypt. Our results demonstrated that the prevalence of astrovirus in children suffering from gastroenteritis was 28%. We found that astrovirus infection is associated with severe dehydration in infected children. Human astroviruses were more frequently detected from developing countries than from industrialized countries. Previous studies showed that the prevalence of astroviruses among Egyptian children aged <3 years was 17% [19]. During our study, co-circulation of classic and novel astrovirus was characterized in children in Egypt. HAstV-4 and HAstV-1 are the most common serotypes identified. Previous studies conducted in Egypt showed that HAstV-1 was the most prevalent serotype of classical HAstVs [19,20]. Novel MLB1 HAstV was also identified in infected children in Egypt. Detection of a novel MLB1 astrovirus from children with diarrhea residing in Egypt agrees with a previous study [20]. Notably, our results showed that a positive sample from children was phylogenetically related to sheep astrovirus genome sequence. This study showed that gender and age have some sort of significant association with astrovirus infection, where males and children ≤1 year of age were more sensitive than females and older children to astrovirus infection [21].

Bats can harbor many different viruses that have a zoonotic potential and may be a natural reservoir for astroviruses. The prevalence rate of astroviruses in collected samples from bats in Egypt was 28.53%, indicating active circulation of AstVs in bats. Although there was a high prevalence rate of astroviruses in bats during our study, all of these animals appeared to be healthy, in alignment with previous findings [13,17]. In line with previous studies, no significant difference was found in AstV detection rate in samples collected from bats between males and females, or adults and juveniles [14]. We identified AstVs in bats with parental relationships to several known AstVs characterized in bats, chickens, dolphins, deer, and cheetahs. High genetic diversity was detected among AstVs sequences obtained from Egyptian bats. This finding is consistent with previous studies performed in different regions in the world [9,10,14,22].

In this study, astroviruses were detected in 26.57% of cloacal swabs, collected from apparently healthy populations of wild birds in Egypt from order Anseriformes, Columbiformes, Gruiformes, Pelecaniformes, and Charadriiformes. We noted that not all of our studied species were positive for astroviruses (Table 1). These species may have been negative as a result of the limited collected samples from each of these species. In contrast to the highly positive rate of astroviruses detected in wild birds in Egypt in our study, previous studies conducted in wild birds in French Guiana, Cambodia, and Hong Kong showed 1.7–4.9 % prevalence of astroviruses [23,24]. Our findings reveal that wild birds are major reservoirs of several types of avastroviruses, and infection with diverse astroviruses is extensively common in wild bird populations. The two strains detected from resident birds, Black winged stilt and Glossy ibis, were phylogenetically related to classical human astroviruses. Until now, there have been no known infections of humans with avian astroviruses. The two previously mentioned species infected with classical human astrovirus feed along the muddy banks of the Nile River. Accordingly, fecal contaminated water may be a source of infection. Previous studies showed a prevalence of human astroviruses in muddy sediment samples and surface water of the Nile River [25,26].

For a long time, it was believed that the transmission of astroviruses had strict species specificity [27]. However, recent evidence showed cross-species transmission and infection of astroviruses. Based on evolutionary analysis of the obtained sequences, results indicated that an interspecies transmission of astroviruses among humans, bats, other mammals, and birds may be potentially occurring.

A limitation of this study was the classification of detected astroviruses based on a partial sequence of RdRp. However, the limited number of capsid sequences available, compared to RdRp sequences, makes consistent classification difficult, especially with some novel viruses incompletely sequenced. This impeded a deeper phylogenetic, evolutionary, and molecular analysis. Further virological and epidemiological studies from a broader host range are needed to determine the pathogenicity and host range of circulating astroviruses. More research on astroviruses is needed, and this family should not be neglected further.

In conclusion, we have characterized astroviruses circulating in humans, bats, and wild birds in Egypt. The close phylogenetic relationship between some avian, bat, and human astroviruses implies the possibility of cross-species transmission among different hosts. Our findings suggest the need for systemic surveillance of different species of mammals and birds.

## Figures and Tables

**Table 1:** Epizootiologic data of astroviruses in humans, bats, and wild birds in Egypt.

Variable	No. Collected Samples (% *)	No. of Astroviruses-Positive Samples (% ^†^)	*p*-Value
***Human***	100	28 (28)	
*Governorate*			
Fayoum	2 (2)	1 (50)	NS
Giza	64 (64)	20 (31.25)	
Cairo	26 (26)	7 (26.92)	
Alexandria	1 (1)	0 (0)	
Aswan	1 (1)	0(0)	
Qaulibia	4 (4)	0 (0)	
Suiz	2 (2)	0(0)	
*Sex*			
Male	66 (66)	15 (22.72)	*p* < 0.05
Female	34 (34)	13 (38.23)	
*Age*			
≤1	71 (71)	25 (35.21)	*p* < 0.05
>1	29 (29)	3 (10.34)	
***Bats***	417	119 (28.53)	
	*Governorate*		
Damietta	116 (27.81)	13 (11.21)	*p* < 0.05
Giza	301 (72.19)	106 (35.22)	
*Species*			
Egyptian Fruit Bat (*Rousettus aegyptiacus*)	288 (69.06)	96 (33.33)	*p* < 0.05
Egyptian Pipistrelle (*Pipistrellus aegyptius*)	93 (22.3)	14 (15.05)	
Egyptian slit-faced bat (*Nycteris thebaica*)	25 (5.99)	6 (24)	
Egyptian tomb bat (*Taphozous perforates*)	11 (2.64)	3 (27.27)	
*Age*			
Adult	407 (97.6)	116 (28.5)	NS
Juvenile	10 (2.4)	3 (30)	
***Sex***			
Male	170 (40.7)	58 (34.11)	NS
Female	131 (31.41)	48 (36.64)	
Unknown	116 (27.82)	13 (11.20)	
***Birds***	301	80 (26.5)	
*Governorate*			
Aswan	79 (26.25)	13 (16.45)	*p* < 0.05
Damietta	113 (37.54)	42 (13.95)	
Fayoum	19 (6.31)	10 (52.63)	
Mersa Matruh	24 (7.97)	4 (16.66)	
Port Said	66 (21.92)	11 (16.66)	
*Species*			
African swamphen (Porphyrio madagascariensis)	5 (1.66)	0 (0)	*p* < 0.05
Cormorant *(Phalacrocorax carbo)*	2 (0.66)	0 (0)	
Squacco heron (*Ardeola ralloides*)	2 (0.66)	1(50)	
Glossy ibis (*Plegadis falcinellus*)	4 (1.33)	1 (25)	
Grey heron (*Ardea cinereal*)	10 (3.32)	3 (30)	
Laughing dove (*Spilopelia senegalensis*)	6 (1.99)	1 (16.66)	
Moorhen (*Gallinula chloropus*)	2 (0.66)	0(0)	
Teal (*Anas crecca*)	65 (21.59)	15 (23.07)	
Wigeon (*Anas Penelope*)	1 (0.33)	1 (100)	
Black-winged stilt (Himantopus himantopus)	9 (2.99)	1 (11.11)	
Coot (*Fulica atra*)	12 (3.98)	2 (16.66)	
Egyptian goose (Alopochen aegyptiacus)	5 (1.66)	1 (20)	
Mallard (*Anas platyrhynchos*)	6 (1.99)	3 (50)	
Namaqua dove (Oena capensis)	4 (1.33)	0 (0)	
Northern shoveler (*Anas clypeat*)	101 (33.55)	26 (25.74)	
Pintail (*Anas acuta*)	44 (14.61)	20 (45.45)	
Purple heron (*Ardea purpurea*)	4 (1.32)	1 (25)	
Spotted redshank (*Tringa erythropus*)	10 (3.32)	2 (20)	
*Habitat*			
Migratory birds	270 (89.70)	74 (27.40)	NS
Resident birds	31 (10.30)	6 (19.35)	

*p*-value was obtained by performing Chi-square test; NS, not significant; * % of total samples collected; ^†^ % of samples in category.

## Supplementary Materials

The following are available online at https://www.mdpi.com/1999-4915/12/5/485/s1, Figure S1: A maximum-likelihood phylogenetic tree of RNA-dependent RNA polymerase gene of the detected astroviruses, Figure S2: A maximum-likelihood phylogenetic tree of RNA-dependent RNA polymerase gene of the detected, Table S1: Pairwise genetic distance calculation among nucleotide sequences of human and bat astroviruses astroviruses in wild bird samples. Astroviruses sequenced specifically for this study are labeled with red circles. In human and bat samples. Human and bat Astroviruses sequenced specifically for this study are labeled with black circles and red squares, respectively. Table S2: Pairwise genetic distance calculation among nucleotide sequences of avian astroviruses.
